# Editorial: Recent advances in gestational diabetes: diagnosis, treatment and prevention

**DOI:** 10.3389/fendo.2026.1773541

**Published:** 2026-01-15

**Authors:** John Punnose, Christian S Göbl, Sarah J Glastras, Andrea Tura

**Affiliations:** 1 Department of Endocrinology, St. Stephen’s Hospital, Delhi, India; 2 Department of Obstetrics and Gynaecology, Medical University of Vienna, Vienna, Austria; 3 Department of Diabetes, Metabolism and Endocrinology, and Kolling Institute, Royal North Shore Hospital, Sydney, NSW, Australia; 4 CNR Institute of Neuroscience, Padova, Italy

**Keywords:** biomarker, diagnosis, gestational diabetes, OGTT, screening

Gestational diabetes mellitus (GDM) is a common metabolic disorder in pregnancy characterized by glucose intolerance first identified in the second or third trimester (1). GDM predisposes pregnant women to several obstetric and perinatal complications and places the mother and infant at risk of long-term metabolic morbidity (2). Traditionally, GDM is diagnosed using an oral glucose tolerance test (OGTT) after 24 weeks of gestation. The current practice of GDM testing is relatively late in pregnancy, potentially limiting the opportunity for early interventions to prevent adverse pregnancy outcomes, especially among high-risk population groups. In fact, there is emerging evidence to suggest deleterious effects of ‘intermediate hyperglycemia’ in early pregnancy, and early therapeutic intervention could potentially reduce several serious neonatal complications (3, 4). These observations emphasize the need for a reliable test to predict or diagnose GDM in early pregnancy.

Several glycemic markers, including glycated hemoglobin (HbA1c) and fasting plasma glucose, serve as potential diagnostic markers for GDM and have been extensively studied (5). Less studied glycemic markers include 1,5-anhydroglucitol (1,5-AG), CD59 (pGCD59), second-trimester glycated albumin, and fructosamine levels (6, 7). Among these biomarkers, only HbA1c seems promising and could be an early marker for GDM. Currently, there is growing interest in identifying non-glycemic biomarkers for GDM prediction in early pregnancy. These biomarkers relate to pathogenetic events in GDM development: especially, insulin resistance and pancreatic β-cell dysfunction, caused by various factors like placental hormones, inflammation, metabolic changes, genetics, and epigenetic changes (8). The non-glycemic biomarkers under evaluation include adipokines, inflammatory and immunological markers, placenta-derived markers, thyroid function and lipid profile markers, hematological markers, and genetic markers (8).

In the present Research Topic, ‘*Recent Advances in Gestational Diabetes: Diagnosis, Treatment, and Prevention*, three articles focused on the association of GDM with non-glycemic biochemical parameters in early pregnancy: serum pancreatic duodenal homeobox-1 (PDX1) gene, ferritin, and bile acids.

PDX1 is a nuclear factor that has a pivotal role in the differentiation of β-cells and is a promoter of the insulin gene expression, thereby increasing the synthesis of insulin and maintaining glucose homeostasis (9, 10). In a prospective study, Zhang et al. assessed serum PDX-1 levels at 8–12 gestational weeks among 231 pregnant Chinese women and assessed their association with GDM. PDX1 in early pregnancy was negatively correlated with fasting and 2h plasma glucose, HOMA-IR, and the triglyceride-glucose (TyG) index, and positively correlated with HOMA-β in mid-pregnancy (P<0.05). The adjusted analysis showed that elevated PDX1 levels in early pregnancy were associated with reduced risks of GDM (adjusted odds ratio, aOR: 0.287, 95% CI 0.130-0.636, P = 0.002). The area under the receiver operating characteristic (ROC) curve of PDX1 in early pregnancy for predicting the occurrence of GDM was 0.616 (P<0.05). The authors concluded that PDX1 has a modest predictive value for GDM, though its addition did not significantly improve the predictive value of conventional GDM risk factors.

Elevated serum ferritin (SF) levels are associated with oxidative stress (OS) and systemic inflammation in various disorders. SF is significantly increasedin early pregnancy among women with GDM and singleton pregnancies in several studies and may serve as a potential biomarker (11, 12). In the present Research Topic, Ni et al. explored the association between SF levels in early pregnancy and the risk of GDM in twin pregnancies. This retrospective cohort study included 882 twin pregnancies (700 dichorionic-diamniotic (DCDA) and 182 monochorionic-diamniotic (MCDA). In MCDA pregnancies, women with GDM had significantly higher mean SF levels compared to womenwithout GDM (101.68 ± 59.72 vs. 79.87 ± 53.11 μg/L, p < 0.05). In MCDA cases, SF >71.4 μg/L wasindependently associated with an increased risk of GDM (aOR = 2.775, 95% CI: 1.191–6.466; p = 0.018), with a significant trend across SF levels (P for trend equal to0.012). The area under the ROC curve of the prediction model of GDM in MCDA pregnancy using SF was 0.77. The authors suggest SF as a potential early biomarker for GDM prediction in MCDA pregnancies. In contrast, no significant association between SF levels and GDM was observed in DCDA pregnancies, suggesting that chorionicity is relevant in the metabolic evaluation of twin gestations.

Lu et al.’s review article suggests a potential association between GDM and bile acids. The primary focus of this review is the role of bile acids in glucose and lipid homeostasis as vital signals that regulate the Farnesoid X receptor (FXR) and Takeda G protein-coupled receptor 5 (TGR5), highlighting their potential as novel therapeutic targets for GDM management. The authors also present evidence supporting bile acids as promising biomarkers for diagnosing and assessing GDM risk. Taurocholic acid and β-muricholic acid exhibit a positive correlation with GDM risk, whereas lithocholic acid, glycodeoxycholic acid, glycoursodeoxycholic acid, and deoxycholic acid demonstrate a negative correlation.

To sum up, the three emerging non-glycemic biomarkers, PDX-1, ferritin, and bile acids, are potential predictors for GDM development in early pregnancy but lack adequate sensitivity and specificity to replace the cumbersome OGTT as a diagnostic test for GDM. Nonetheless, there remains a strong need for a reliable, simple biomarker to predict the development of GDM in early pregnancy. Early identification could reduce the period of exposure to fetal hyperglycemia through targeted prevention and therapeutic strategies, yet the heterogeneity in the aetiopathogenesis, phenotypical characteristics, and genetic architecture of GDM women remains a significant challenge (13).
